# Supplementary material for: Antibody and DNA sensing pathways converge to activate the inflammasome during primary human macrophage infection
Source: EMBO J. 2019 Aug 29;38(21):e101365. doi: 10.15252/embj.2018101365 (PMC6826209; doi:10.15252/embj.2018101365)

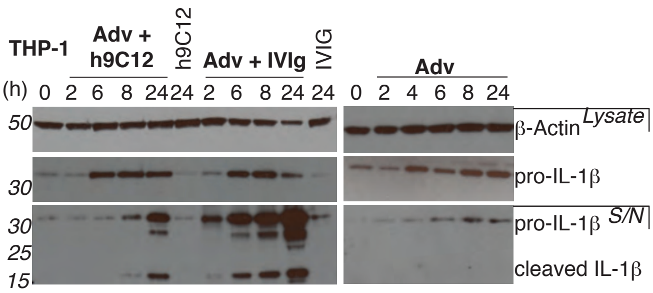

ECL blots and scanned films
Key to handwritten legends:
AdV + IgG1 = AdV + 9C12
AdV + Sanq = AdV + IVIg
pI:C/Nig etc - cropped from final blots

On original blots below:
30 kDa marker mislabelled as 35,
50 kDa marker mislabelled as 55

Figure 1C - β–Actin - Cell Lysate

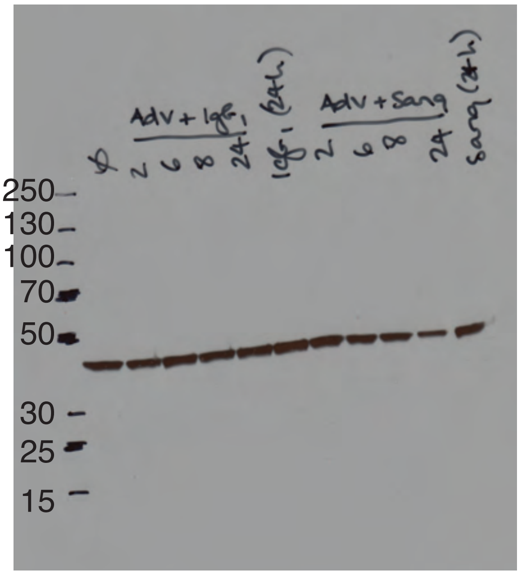

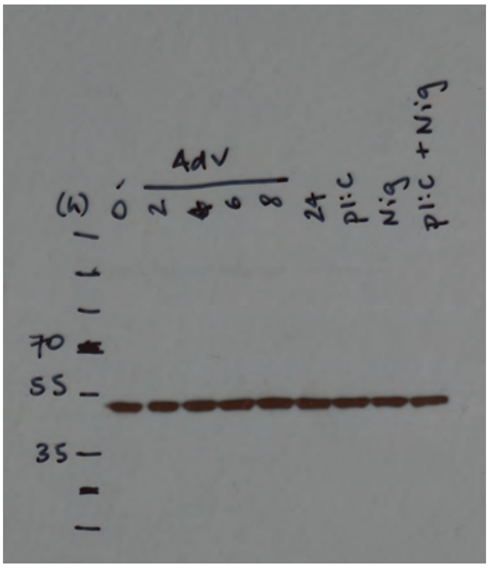

Figure 1C - pro IL-1β - Cell Lysate

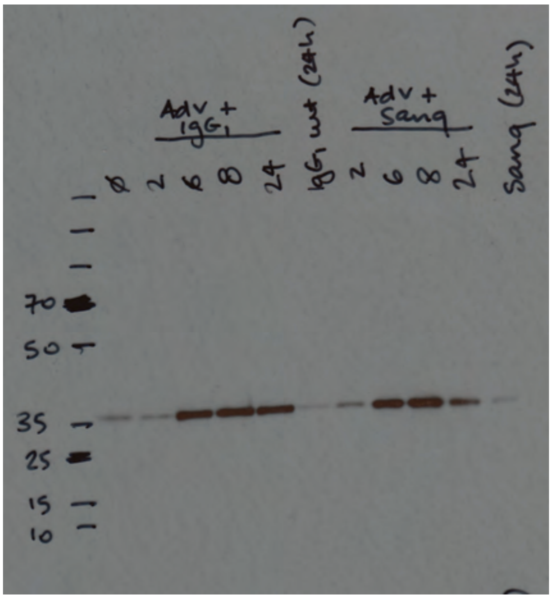

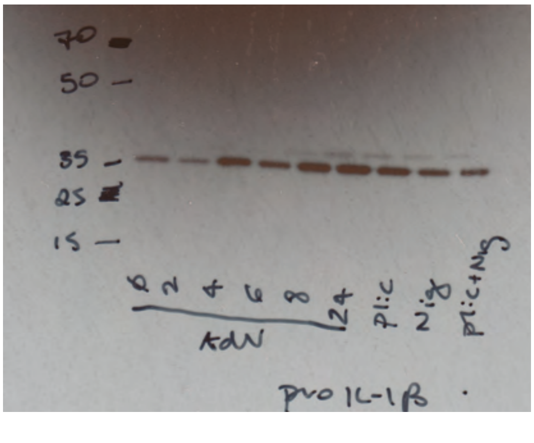

Figure 1C - pro IL-1β and cleaved IL-1β Supernatants

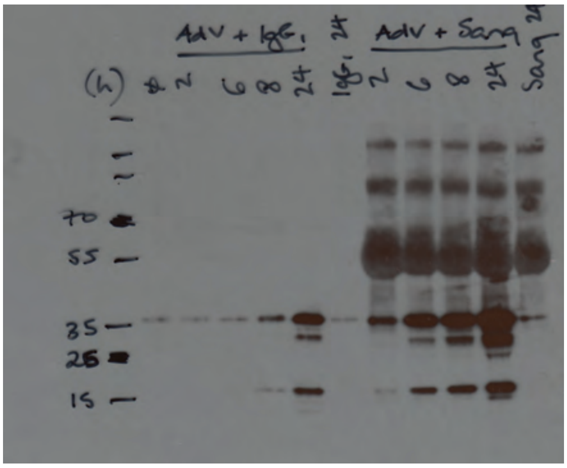

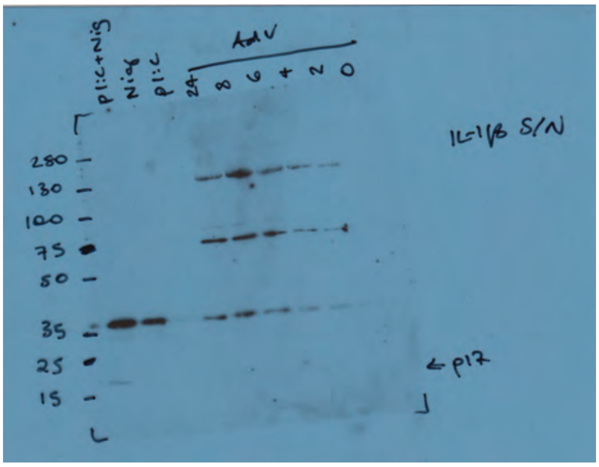

Supplement: Supplementary file 1 — Source Data for Figure 1 [file EMBJ-38-e101365-s001.pdf]
