# Supplementary figures and images for: Antibody and DNA sensing pathways converge to activate the inflammasome during primary human macrophage infection
Source: EMBO J. 2019 Aug 29;38(21):e101365. doi: 10.15252/embj.2018101365 (PMC6826209; doi:10.15252/embj.2018101365)

Figure 4D

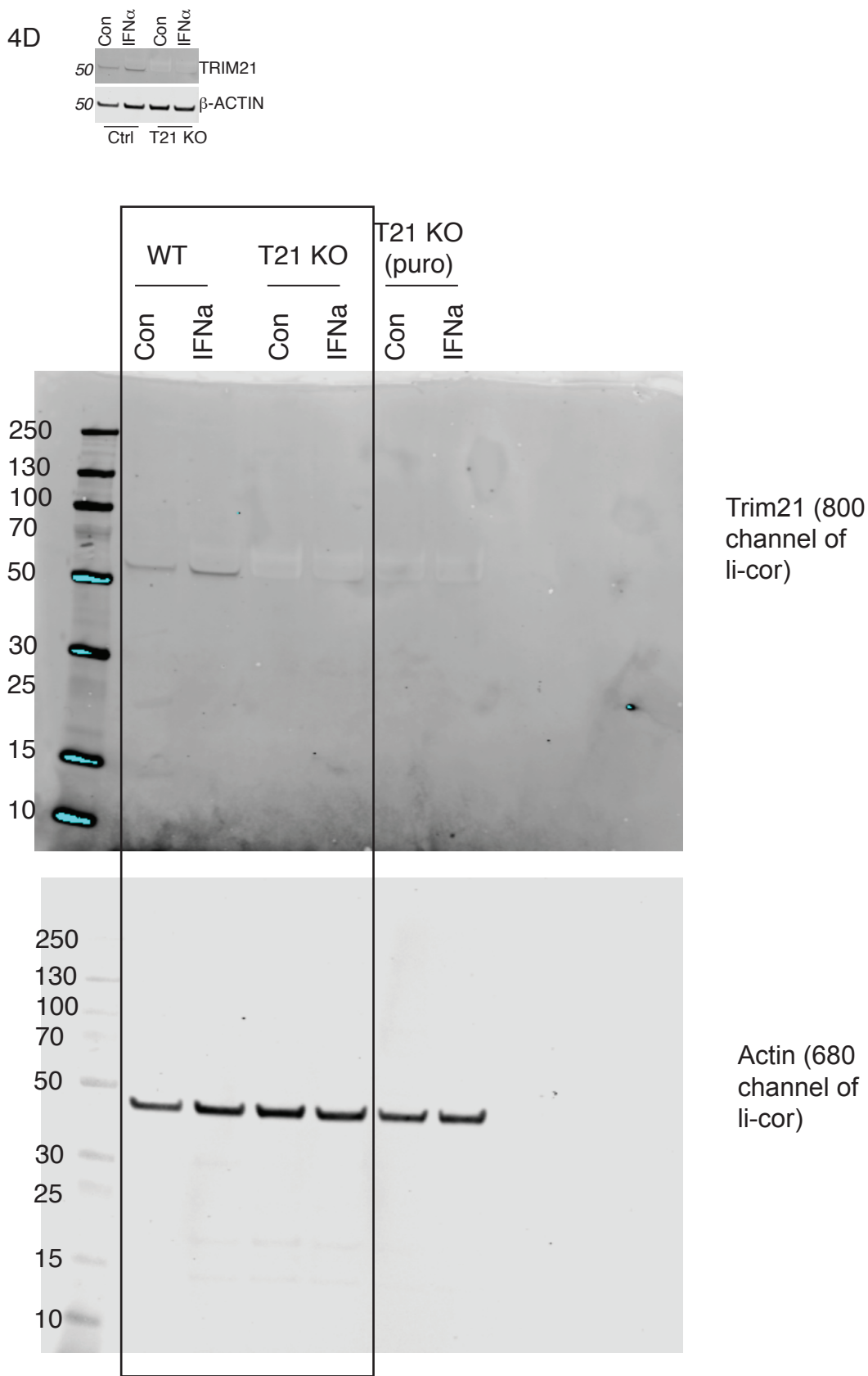

Supplement: Supplementary file 2 — Source Data for Figure 4 [file EMBJ-38-e101365-s002.pdf]
