# Supplementary material for: Antibody and DNA sensing pathways converge to activate the inflammasome during primary human macrophage infection
Source: EMBO J. 2019 Aug 29;38(21):e101365. doi: 10.15252/embj.2018101365 (PMC6826209; doi:10.15252/embj.2018101365)

Figure 5B

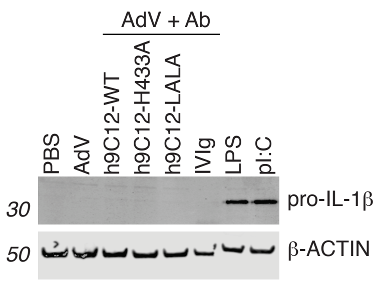

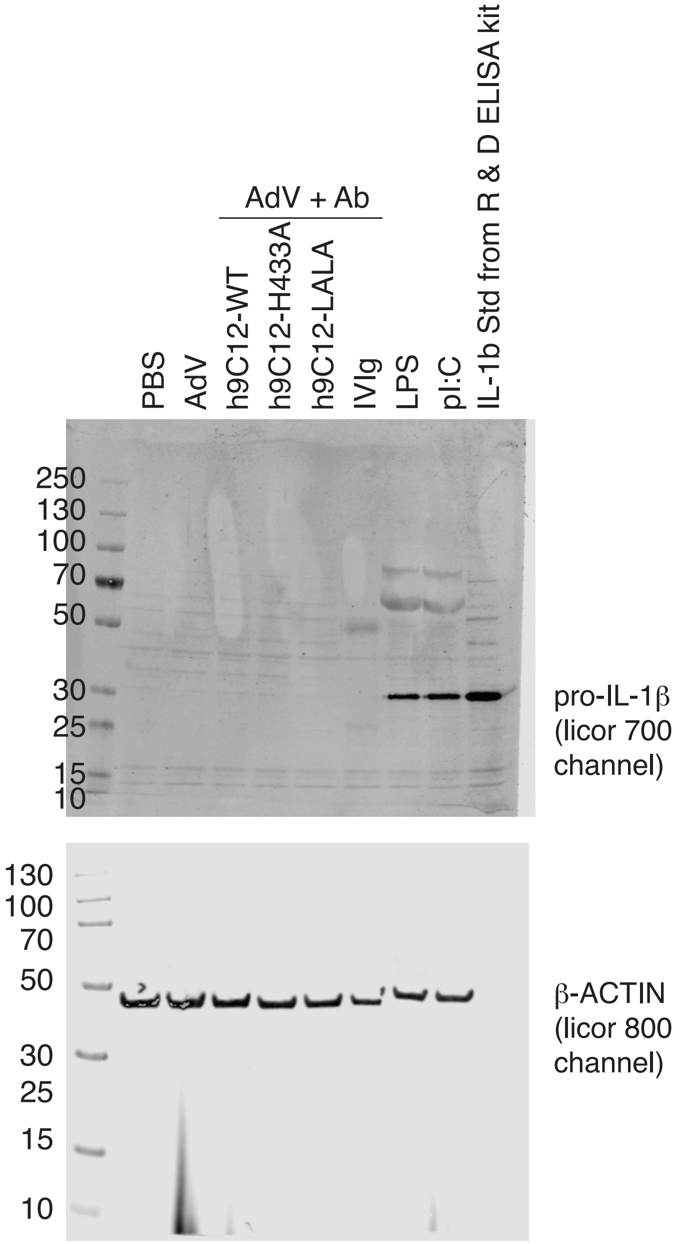

Figure 5D

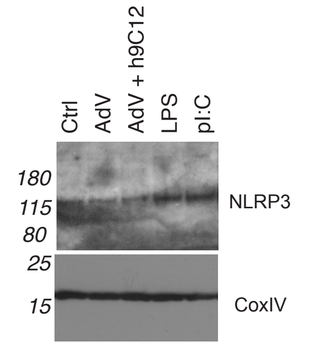

ECL - film scanned in

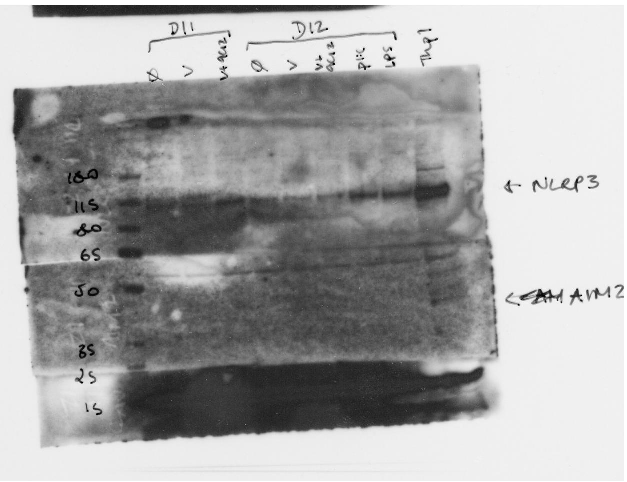

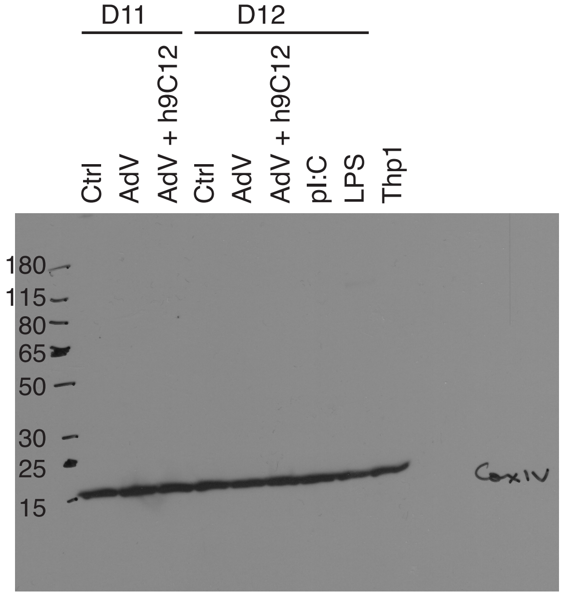

Supplement: Supplementary file 3 — Source Data for Figure 5 [file EMBJ-38-e101365-s003.pdf]
