# Supplementary material for: Antibody and DNA sensing pathways converge to activate the inflammasome during primary human macrophage infection
Source: EMBO J. 2019 Aug 29;38(21):e101365. doi: 10.15252/embj.2018101365 (PMC6826209; doi:10.15252/embj.2018101365)
Supplement: Supplementary file 4 — Source Data for Figure 7 [file EMBJ-38-e101365-s004.pdf]

Figure 7B

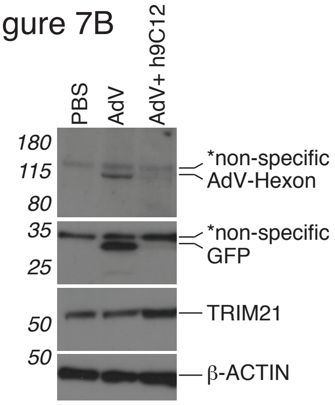

Figure 7C

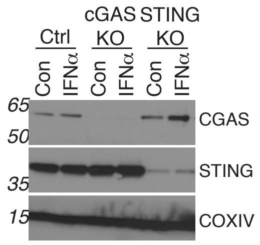

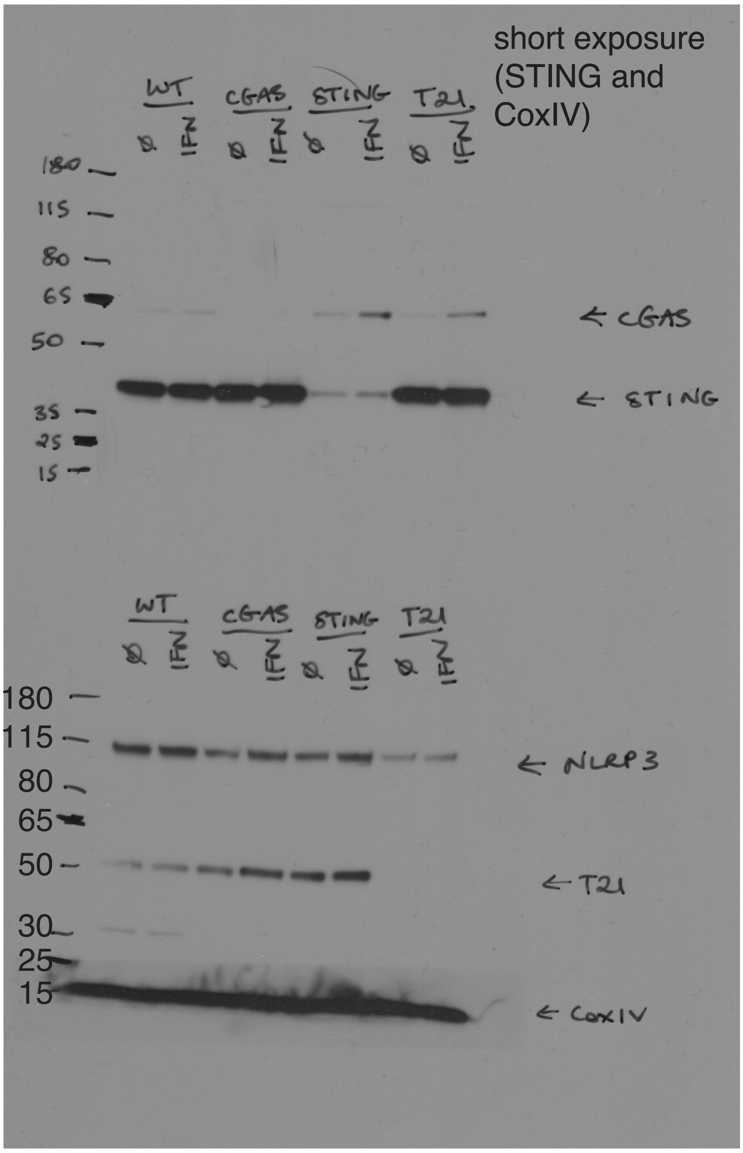

Membranes were cut after transfer and incubated with indicidual antibodies, ECL and films scanned.

Key:

Adv = v

AdV + 9C12 = V + WT

V + Sanq (cropped from final image)

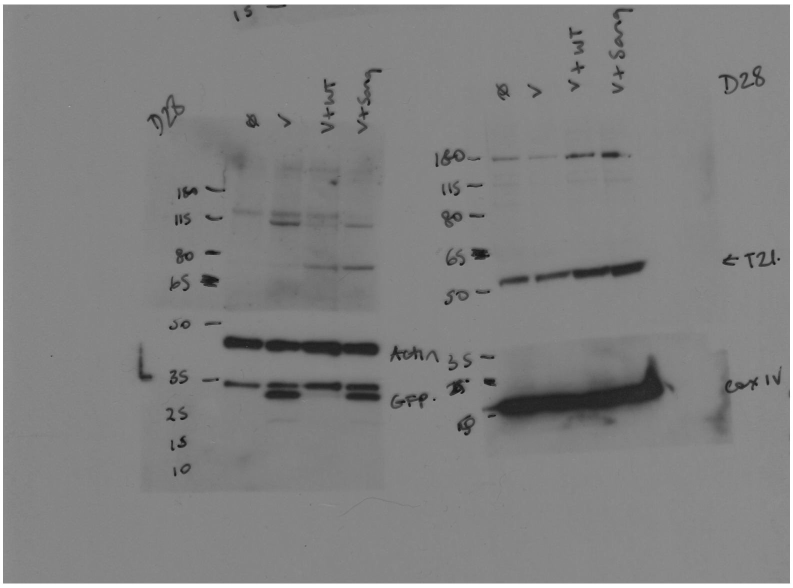

On original blots below:

30 kDa marker mislabelled as 35

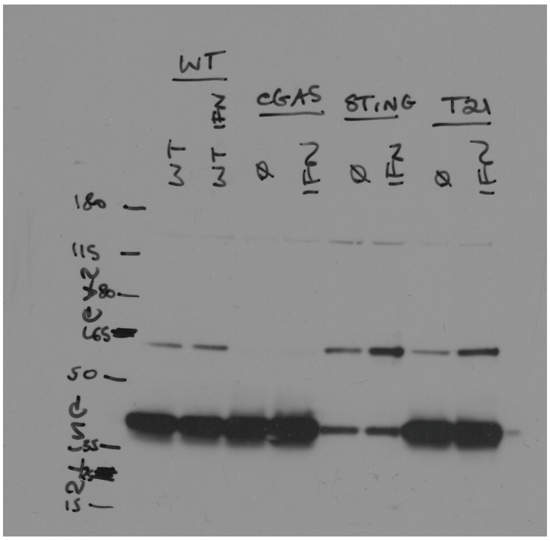

long exposure (cGAS)

cGAS

STING
